# Antiviral Efficacy and Host Innate Immunity Associated with SB 9200 Treatment in the Woodchuck Model of Chronic Hepatitis B

**DOI:** 10.1371/journal.pone.0161313

**Published:** 2016-08-23

**Authors:** Kyle E. Korolowicz, Radhakrishnan P. Iyer, Stefanie Czerwinski, Manasa Suresh, Junming Yang, Seetharamaiyer Padmanabhan, Anjaneyulu Sheri, Rajendra K. Pandey, Jeffrey Skell, Judith K. Marquis, Bhaskar V. Kallakury, Robin D. Tucker, Stephan Menne

**Affiliations:** 1 Department of Microbiology & Immunology, Georgetown University Medical Center, Washington, DC, 20057, United States of America; 2 Spring Bank Pharmaceuticals, Inc., Suite S-7, 113 Cedar Street, Milford, MA, 01757, United States of America; 3 Department of Pathology, Georgetown University Medical Center, Washington, DC, 20057, United States of America; 4 Department of Comparative Medicine, Georgetown University Medical Center, Washington, DC, 20057, United States of America; Indiana University, UNITED STATES

## Abstract

SB 9200, an oral prodrug of the dinucleotide SB 9000, is being developed for the treatment of chronic hepatitis B virus (HBV) infection and represents a novel class of antivirals. SB 9200 is thought to activate the viral sensor proteins, retinoic acid-inducible gene 1 (RIG-I) and nucleotide-binding oligomerization domain-containing protein 2 (NOD2) resulting in interferon (IFN) mediated antiviral immune responses in virus-infected cells. Additionally, the binding of SB 9200 to these sensor proteins could also sterically block the ability of the viral polymerase to access pre-genomic RNA for nucleic acid synthesis. The immune stimulating and direct antiviral properties of SB 9200 were evaluated in woodchucks chronically infected with woodchuck hepatitis virus (WHV) by daily, oral dosing at 15 and 30 mg/kg for 12 weeks. Prolonged treatment resulted in 2.2 and 3.7 log_10_ reductions in serum WHV DNA and in 0.5 and 1.6 log_10_ declines in serum WHV surface antigen from pretreatment level with the lower or higher dose of SB 9200, respectively. SB 9200 treatment also resulted in lower hepatic levels of WHV nucleic acids and antigen and reduced liver inflammation. Following treatment cessation, recrudescence of viral replication was observed but with dose-dependent delays in viral relapse. The antiviral effects were associated with dose-dependent and long-lasting induction of IFN-α, IFN-β and IFN-stimulated genes in blood and liver, which correlated with the prolonged activation of the RIG-I/NOD2 pathway and hepatic presence of elevated RIG-I protein levels. These results suggest that in addition to a direct antiviral activity, SB 9200 induces antiviral immunity during chronic hepadnaviral infection *via* activation of the viral sensor pathway.

## Introduction

Infection with hepatitis B virus (HBV) is responsible for approximately 1.2 million deaths per year worldwide due to HBV-associated liver diseases, including hepatic cirrhosis and hepatocellular carcinoma (HCC) [1]. Current antiviral therapies for chronic hepatitis B (CHB) are limited to nucleos(t)ide analogs and (pegylated) interferon-alpha (IFN-α). Prolonged administration of nucleos(t)ide analogs reduces viral load and improves the long-term outcome of CHB, but rarely leads to a cure [2]. Use of these antivirals is further limited due to the emergence of drug-resistant variants during treatment, the risk of relapse upon treatment discontinuation, and unwanted side effects [2]. Long-term IFN-α administration is also associated with treatment-limiting adverse effects and variability in treatment response, but the rate of durable HBV surface antigen loss is higher than with nucleos(t)ide analogs although still only occurs in <10% of patients [2]. There is therefore an urgent need for improvements in therapeutic strategies for inducing functional cure of CHB.

The Eastern woodchuck (*Marmota monax*) is naturally infected with the woodchuck hepatitis virus (WHV), a hepadnavirus which is genetically closely related to human HBV [3]. Neonatal WHV infection parallels the main route of human (vertical) transmission for chronic HBV infection and displays a disease course similar to that in HBV-infected patients. Thus, chronic WHV infection in woodchucks is a fully immunocompetent model for studying CHB and HBV-induced HCC, and woodchucks have extensively been used to evaluate efficacy and safety of current and new HBV therapeutics [3]. The recent comparison of hepatic transcriptional profiles in woodchucks and humans with acute self-limiting and chronic hepadnaviral infections identified important parallels in the antiviral immune responses induced by WHV and HBV [4]. In addition, these transcription signatures have facilitated the characterization of antiviral immune responses in woodchucks to treatment with GS-9620, a toll-like receptor 7 agonist [5] and IFN-α [6]. As these studies have established the translational value of this animal model for CHB, woodchucks were used to evaluate the antiviral efficacy of SB 9200.

SB 9000 is a small molecule nucleic acid hybrid (a dinucleotide) with antiviral activity against HBV [7, 8], which was further elaborated into an orally bioavailable prodrug compound, SB 9200 [9, 10]. In addition to the direct antiviral property, the host immune stimulating activity of SB 9200 is thought to induce endogenous IFN *via* the activation of the viral sensor proteins, retinoic acid-inducible gene 1 (RIG-I) and nucleotide-binding oligomerization domain-containing protein 2 (NOD2). Activation is believed to occur by binding of SB 9200/SB 9000 to RIG-I and NOD2 at their nucleotide binding domain. Both cytosolic proteins usually recognize signature patterns of foreign RNA such as the pathogen-associated molecular pattern (PAMP). Once PAMP is recognized, RIG-I and NOD2 become activated resulting in the induction of the IFN signaling pathway and the production of type I and III IFNs, as well as the subsequent induction of interferon-stimulated genes (ISGs), pro-inflammatory cytokines and antiviral immune cells [11, 12]. The direct antiviral activity of SB 9200/SB 9000 is thought to inhibit the synthesis of viral nucleic acids by steric blockage of the viral polymerase, similar to the mechanism recently described for HBV [12]. The blockage, which is independent of the IFN signaling pathway, may be achieved by binding of SB 9200/SB 9000 with RIG-I and NOD2 that associate with viral RNA and that in turn prevents the polymerase from engaging with the pre-genomic (pg) RNA template for viral replication. Although no data currently exist that directly support the assumed mechanism, preliminary *in vitro* findings indicate that SB 9200/SB 9000 induces rapid translocation of RIG-I on a double-stranded RNA template and that SB 9000 binds to RIG-I in a dose-dependent way (Radhakrishnan P. Iyer; personal communication). Thus, dose-dependent binding of SB 9000/9200 to RIG-I (and NOD2) and consequent rapid shuttling of sensor proteins on a nucleic acid template (i.e., pgRNA) could displace the viral polymerase from accessing the pgRNA for replication. SB 9200 has been intensively tested *in vitro* and *in vivo* against HBV [8, 9]. SB 9200 is further antiviral efficacious against hepatitis C virus (HCV) [13], and a Phase I evaluation of SB 9200 in humans for therapy of HCV was recently completed [14]. Here we report a single agent efficacy study in which the host immune stimulating and direct antiviral activities of SB 9200 at two separate doses were evaluated in woodchucks with chronic WHV infection. The results show that oral administration of SB 9200 for 12 weeks at two select doses reduced viral markers in serum and liver that were associated with (or were a result of) the induction of host antiviral immune responses.

## Materials and Methods

### Investigational drug

SB 9200, an oral prodrug of SB 9000, was manufactured by Spring Bank Pharmaceuticals, Inc. (Milford, MA) and the structure and antiviral characteristics of SB 9200/SB 9000 have previously been described [7, 10]. Doses of SB 9200 were dry mixed with woodchuck diet powder (Dyets, Bethlehem, PA), the blended drug material suspended in ultrapure water, and orally administered to woodchucks within ½ hour after preparation.

### Repeat dose study in woodchucks

The animal protocol and all procedures involving woodchucks were approved by the IACUC of Georgetown University and adhered to the national guidelines of the Animal Welfare Act, the Guide for the Care and Use of Laboratory Animals, and the American Veterinary Medical Association. Woodchuck were anesthetized by intramuscular injection of ketamine (50 mg/kg) and xylazine (5 mg/kg). Prior to euthanasia, woodchucks were anesthetized as described above and euthanized by an overdose of Beuthanasia-D solution (80–100 mg/kg) administered by intracardiac injection, followed by bilateral intercostal thoracotomy. Woodchucks used in this study were born in captivity and infected at 3 days of age with WHV. Chronically infected animals were confirmed positive for serum WHV DNA and WHV surface antigen (WHsAg) and had undetectable antibodies against WHsAg (anti-WHs) at approximately one year post-infection. Absence of liver tumors in woodchucks with low gamma-glutamyl transferase (GGT) was confirmed by ultrasonography. Chronic WHV carrier woodchucks were assigned and stratified by gender, body weight, and by pretreatment serum markers (WHV DNA and WHsAg loads and serum GGT and sorbitol-dehydrogenase (SDH) levels) into two groups (n = 5 each). Woodchucks were treated once daily, orally with either a low dose (15 mg/kg) or a high dose (30 mg/kg) of SB 9200 for 12 weeks. As inclusion of a placebo-treated control group was not feasible due to the paucity of WHV-infected woodchucks, the treatment effects mediated by SB 9200 at two separate doses were evaluated by changes in viral and host parameters from pretreatment level which served as the control for comparison. For select assays, blood and liver samples from five age-matched, treatment-naïve chronic WHV carrier woodchucks were included for comparison of basal expression levels of immune response genes in untreated animals with pretreatment levels in SB 9200 treated animals.

### Pharmacokinetic analysis of SB 9000

Plasma levels of SB 9000, the active moiety of the prodrug SB 9200, in samples collected at pretreatment and then bi-weekly throughout the study at a single time-point of approximately 2 hours post-dose were evaluated as follows: A 100 μL aliquot of plasma was extracted by solid phase method (Phenomenex Strata X-AW 33 μm, 10 mg bed) and SB 9000 quantified by liquid chromatography using a Waters XBridge C18, 50 x 2.1 mm, 3.5 μm particle size column with a mixture of water/formic (1000:1, v/v; mobile phase A) and methanol/acetonitrile/formic acid (500:500:1, v/v/v; mobile phase B) under gradient conditions (650 μL/minute flow rate). Detection of the analyte was achieved using an AB Sciex API 5000 triple quadrupole LC MS/MS system operating in positive ESI (TurboIonSpray®) mode.

### Serum WHV parameters

Depending on the serum concentration, WHV DNA was quantified weekly by either dot blot hybridization or real-time PCR assay as described [15]. Serum levels of WHsAg and anti-WHs were measured weekly by WHV-specific enzyme immunoassays as described [16].

### Hepatic WHV parameters

Hepatic levels of WHV nucleic acids were determined in liver biopsy samples collected at pretreatment (week -1), during treatment (week 6), at the end of treatment (week 12), and at the end of the study (week 20). WHV RNA was measured by Northern blot hybridization and WHV DNA replicative intermediates (RI) and WHV covalently-closed circular (ccc) DNA were determined by Southern blot hybridization as described [15]. Liver was imunostained with an antibody against WHV core antigen (WHcAg) using a 1:350 dilution. Liver was further immunostained with a cross-reactive antibody against RIG-1 (Origene Technologies, Rockville, MD) using a 1:125 dilution. The immunohistochemistry (IHC) scores for WHcAg and RIG-I expression in liver were derived from the mean of the stained hepatocyte percentage score combined with the mean of the staining intensity score. A composite IHC score of 0 indicates absence of WHcAg or RIG-I staining in all hepatocytes (0%) whereas 8 indicates presence of strong WHcAg or RIG-I staining in 81–100% of hepatocytes. Specifically, the percentage of WHcAg or RIG-I stained hepatocytes was scored on a 0–5 scale, where 0 = 0%, 1 = 1–20%, 2 = 21–40%, 3 = 41–60%, 4 = 61–80%, and 5 = 81–100% of cells stained. The intensity of WHcAg or RIG-I staining was scored on a 0–3 scale, where 0 = no staining, 1 = weak staining, 2 = moderate staining, and 3 = strong staining. The liver histology score was derived from the mean of the lobular sinusoidal hepatitis score combined with the mean of the portal hepatitis score (n = 1–5 portal tracts examined). The composite histology was scored on a 0–6 scale, where 0 = absent hepatitis, >0–2 = mild hepatitis, >2–4 = moderate hepatitis and >4 = marked to severe hepatitis.

### Host immune response parameters

Immune responses associated with treatment were determined by changes in RNA transcript levels of IFN-α, IFN-β, IFN-γ induced protein 10 (IP-10 or CXCL10), interleukin 6 (IL-6), interferon-induced 17 kDa protein (ISG15), and 2'-5'-oligoadenylate synthetase 1 (OAS1) in blood and liver using PCR. Briefly, whole blood was collected into PAXgene blood tubes (Qiagen, Redwood City, CA) at pretreatment (week -1 and T_0_), during treatment (weeks 6 and 12), and during follow-up (week 18) and stored at -70°C until use. Total RNA was isolated with on-column DNase I digestion using the PAXgene Blood miRNA Kit (Qiagen). Total RNA was further isolated from liver biopsy samples collected as indicated above using the RNeasy Mini Kit (Qiagen) with on-column DNase I digestion using RNase-Free DNase (Qiagen). Following reverse transcription of mRNA with the High Capacity cDNA Reverse Transcription Kit (Applied Biosystems) using oligo(dT), complementary (c) DNA samples were amplified on a 7500 Real Time PCR System instrument (Applied Biosystems) using TaqMan Gene Expression Master Mix (Applied Biosystems) and woodchuck-specific primers and probes (Table 1). Woodchuck 18S rRNA expression was used to normalize target gene expression. Transcription levels of target genes were calculated as a fold-change relative to pretreatment level at week -1 (liver) or at T_0_ (blood) using the formula 2^-ΔΔ*Ct*^. Samples from a subset of woodchucks of the low and high dose groups from which liver biopsies could be collected at all time points during the study were analyzed for expression changes of genes involved in the RIG-I/NOD-2 pathway induced by SB 9200, including RIG-I, NOD2, stimulator of IFN genes (STING; also known as transmembrane protein 173 or TMEM173), and IFN regulatory factor 3 (IRF3).

**Table 1:** Oligonucleotides used for analyses of RIG-I/NOD2 pathway and host innate immune responses in blood and liver.

Gene	Primers and Probe	Sequence
IFN-α	F	5'-CTCAAGCTGTTGCTGTCCTC-3'
	R	5'-CTTCTGGGTGCTGAAGAGGT-3'
	P	5'-CCAGATGACCCAGCAGATCCTCA-3'
IFN-β	F	5'-GAATGAAAGGCCTGCAGAGT-3'
	R	5'-GGATGTTTGATCTCCTTGGG-3'
	P	5'-CTTGAAGTCCATCCTGTCACTGAGGC-3'
CXCL10	F	5'-AAAAAGAGCGGGGAGAAGAG-3'
	R	5'-GGAGCCCTTTTAGACCTTTCAT-3'
	P	5'-TCCAGAATCTAAAGCCATCAAGA-3'
IL-6	F	5'-CCATGCAACTCATCTTGAGC-3'
	R	5'-ATGCCCATGAACCAATAAGC-3'
	P	5'-ATTTCCTGCAGTTCACCC-3'
ISG15	F	5'-CTGTTCTGGCTGAGCTTCG-3'
	R	5'-GCAGGTTCAGAAACACAGTGC-3'
	P	5'-GGGAGTATGGACTCACCCCT-3'
OAS1	F	5'-AGTTCACGATGGTCCAATCC-3'
	R	5'-GTGCCAGGGCATCAAAAG-3'
	P	5'-GCTTCGTGCTGAGTTCCTCT-3'
	F	5’-ACTTTGGATGGTGCCTGAAA-3’
RIG-I	R	5’-AGTGGGATACGGCGTAGAA-3’
	P	5’-TTTCGTTGTAGGACTGGGTTGGTCC-3’
	F	5’-CTGTACGGCTTATGCCATCTT-3’
NOD2	R	5’-ACATATCTGTGGTGGTCTTTGG-3’
	P	5’-TCATGGATGGTGTCCAGATGCCA-3’C
	F	5’-CTATGAGCTTCTGGAGAATGGG-3’
STING	R	5’-CTCTGCCATCCTGTGACATG-3’
	P	5’-CTGTGTCCTGGAGTATGCCACCC-3’
	F	5’-TAGGCAGCTTAGTACACGTTTC-3’
IRF3	R	5’-GACACTATCTGGACAGCCAATAA-3’
	P	5’-TAGCCCATCACTCCTCTGTCTGTCA-3’
	F	5’- GTAACCCGTTGAACCCCATT-3’
18S rRNA	R	5’- GGGACTTAATCAACGCAAGC-3’
	P	5’- GCAATTATTCCCCATGAACG-3’

F: forward primer; R: reverse primer; P: probe.

### Drug safety parameters

Various measurements (body weight, body temperature, clinical chemistry, and hematology) were obtained weekly to monthly for monitoring drug safety.

### Statistical analyses

All parameters were compared to the values at pretreatment and between both dose groups using unpaired Student’s *t*-test with equal variance. *P* values of <0.05 were considered statistically significant.

## Results

### SB 9200 treatment of chronic WHV carrier woodchucks was well tolerated and induced dose-dependent suppression of serum viremia

**Fig 1:**
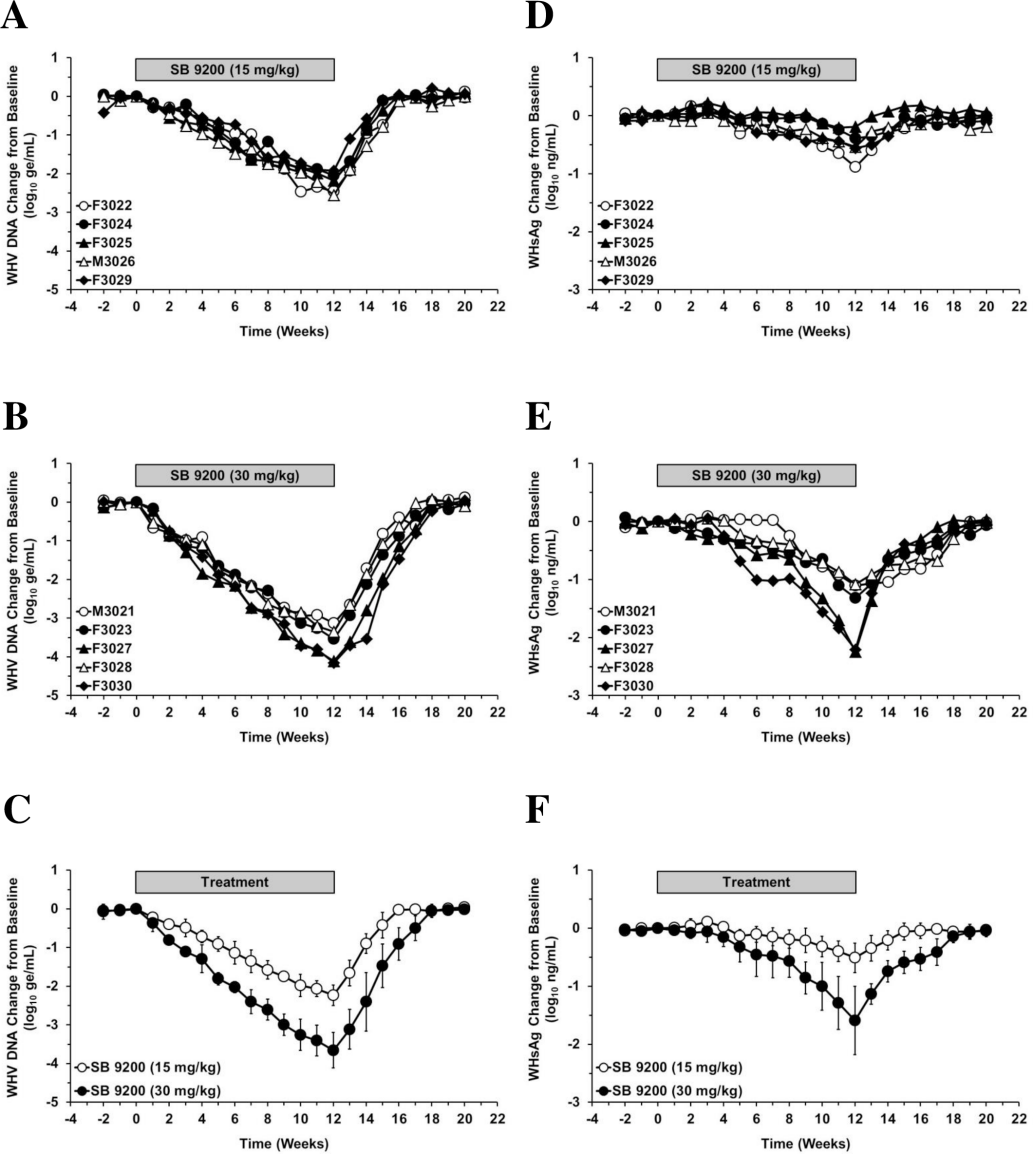
SB 9200 treatment induces dose-dependent, transient suppression of serum viremia and antigenemia. Changes in WHV DNA (**left panels**) and WHsAg levels (**right panels**) relative to T_0_ (pretreatment baseline) during daily, oral treatment with SB 9200 for 12 weeks in individual woodchucks administered a low (**A, D**) or high dose (**B, E**) and mean of each group (**C, F**). At T_0_, mean WHV DNA levels were 4.78x10^10^ and 5.27x10^10^ genomic equivalents/mL serum and mean WHsAg levels were 3.30x10^5^ and 4.32x10^5^ ng/mL serum in the low or high dose groups, respectively. Error bars represent the standard error of the mean.

SB 9200 treatment for 12 weeks was well tolerated, and there were no signs of overt toxicity based on gross observations, body weights, body temperatures, hematology, or clinical chemistry (data not shown), and no mortality was observed during the study. SB 9200 treatment induced reductions in serum WHV DNA from pretreatment level in all woodchucks which were more pronounced and sustained in animals administered the higher dose (Fig 1A–1C). Reductions in mean viral load during the 12-week treatment period occurred gradually, and WHV DNA declined by approximately 1 log_10_ every 3 or 6 weeks, respectively, in the high and low dose groups. At the end of treatment, the maximum mean reduction of WHV DNA in the low and high dose groups was 2.2 or 3.7 log_10_, respectively (Fig 1C). Maximum viral load reductions in two woodchucks of the high dose group were 4.1 and 4.2 log_10_ (Fig 1B). After the end of treatment, rebound in viral load was observed in all woodchucks and WHV DNA increased to pretreatment level during the 8-week follow-up period. In woodchucks treated with the lower dose, WHV DNA returned to pretreatment level within 3–5 weeks following the end of treatment but animals administered the higher dose had a delay in relapse by 2–4 weeks as pretreatment level was reached 5–7 weeks after the end of treatment. Mean viral load in the low and high dose groups during weeks 1–15 or weeks 1–17, respectively, were significantly reduced compared to pretreatment level (all *p*<0.05). Furthermore, the mean viral load of the high dose group during weeks 2–17 was significantly lower than in the low dose group (all *p*<0.05).

### SB 9200 treatment resulted in dose-dependent reduction in serum antigenemia without seroconversion

SB 9200 administration caused dose-dependent reductions in serum WHsAg from pretreatment level in all woodchucks (Fig 1D and 1E). Declines in antigenemia were more marked and durable in animals treated with the higher dose. The maximum mean reduction of WHsAg observed in the low and high dose groups at the end of treatment was 0.5 or 1.6 log_10_, respectively (Fig 1F). Maximum antigen load reductions in two woodchucks of the high dose group were >2.1 log_10_ (Fig 1E). Following the end of treatment, rebound in antigen load to pretreatment level was noted, and WHsAg returned to pretreatment level within 1–5 weeks in woodchucks of the low dose group. In animals administered the higher dose, a 1–7 week delay in antigen rebound was observed, and pretreatment level was reached 6–8 weeks after the end of treatment. Mean antigen load was significantly reduced in the low and high dose groups during weeks 10–14 or weeks 9–16, respectively, when compared to the pretreatment level (all *p*<0.05), and in the high dose group during weeks 11–16, when compared to the low dose group (all *p*<0.05). As SB 9200 administration at the doses and duration applied was unable to produce complete loss of detectable WHsAg (and WHV DNA) in woodchucks, seroconversion to anti-WHs antibodies was not observed (data not shown).

### SB 9200 treatment resulted in dose-dependent increases in total plasma exposure of SB 9000

**Fig 2:**
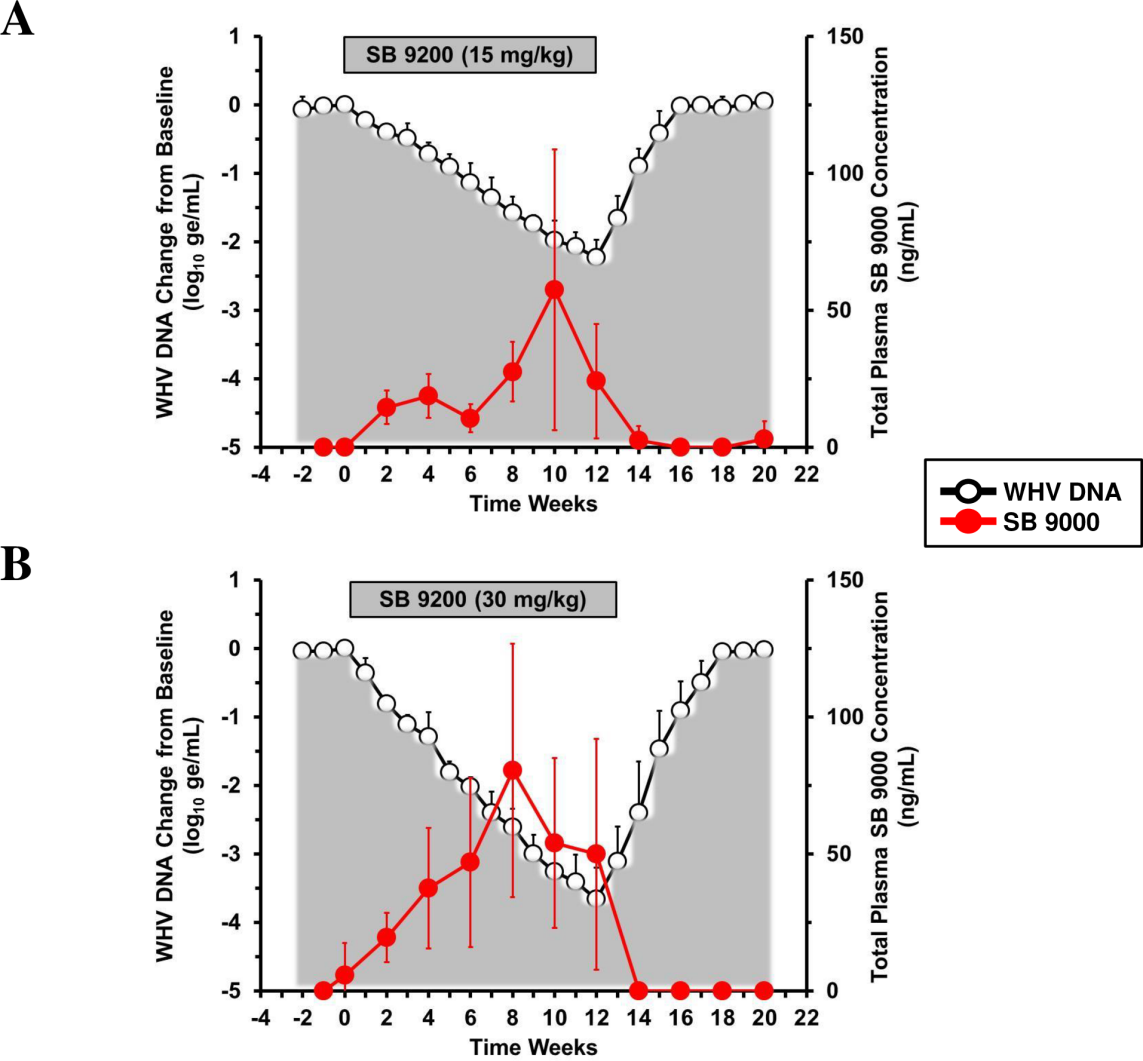
SB 9200 treatment results in dose-dependent increases in total plasma exposure of SB 9000. Changes in mean plasma levels of SB 9000 during daily, oral treatment with SB 9200 for 12 weeks in woodchucks administered a low (**A**) or high dose (**B**). Changes in mean serum WHV DNA relative to T_0_ is plotted on the left y-axis. Error bars represent the standard error of the mean. Because plasma values represent single time-point values at 2 hours post-dosing, it is likely that the peak individual plasma levels do not represent the actual C*max* since the T*max* in individual woodchucks could vary. Peak levels in individual woodchucks were between 25–150 and 75–170 ng/mL in the low and high dose groups, respectively.

The plasma level of SB 9200 was estimated by LC/MS analysis as the SB 9000 level since the prodrug (SB 9200) is rapidly converted to the active moiety (SB 9000) by esteratic hydrolysis [10]. Overall, the plasma exposure of SB 9200 was dose-dependent and correlated well with the observed reductions in viremia (and antigenemia) in both groups (Fig 2). The plasma exposure of SB 9200 was significantly increased during treatment in both groups, when compared to the pretreatment level (*p*<0.05), and in the high dose group at weeks 6 and 8, when compared to the low dose group (all *p*<0.05).

### SB 9200 treatment induced dose-dependent reduction in hepatic levels of WHV nucleic acids

**Fig 3:**
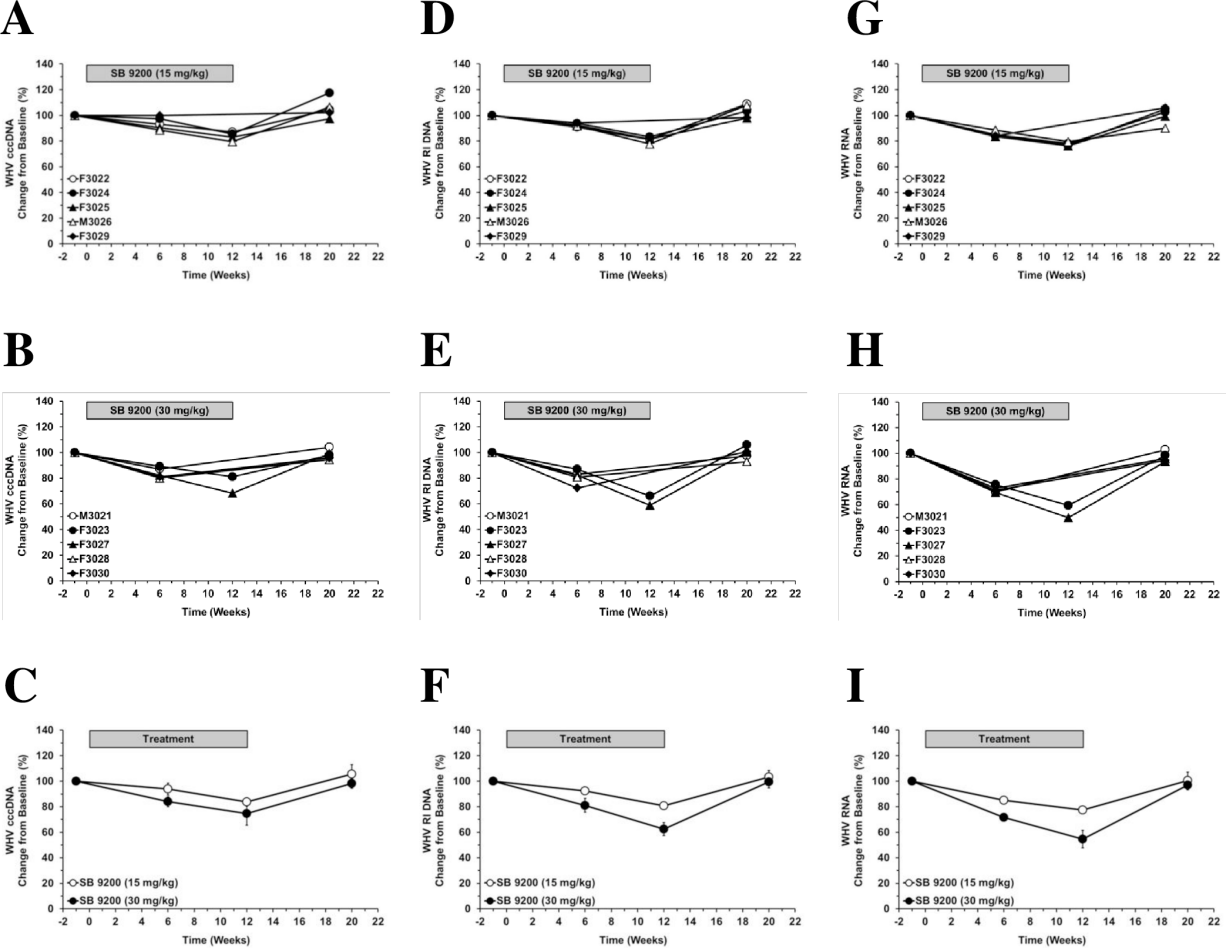
SB 9200 treatment induces dose-dependent, transient reduction in hepatic WHV nucleic acids. Changes in mean hepatic levels of WHV cccDNA (**left panels**), WHV RI DNA (**middle panels**) and WHV RNA (**right panels**) relative to week -1 (pretreatment baseline) in response to SB 9200 treatment at a low (**A, D, G**) or high dose (**B, E, H**) and mean of each group (**C, F, G**). At week -1, WHV DNA RI levels were 1830.9 and 1965.7 pg/μg total DNA and WHV RNA levels were 42.9 and 44.3 pg/μg total RNA in the low and high dose groups. Error bars represent the standard error of the mean.

Compared to pretreatment level, SB 9200 administration induced dose-dependent reductions in the levels of hepatic WHV cccDNA, WHV DNA RI, and WHV RNA (Fig 3). Although liver biopsies could not be collected from all woodchucks at the end of treatment, the declines in these viral markers correlated well with the reductions in serum viremia and antigenemia (compare Fig 1 and Fig 3). At the end of treatment, the maximum reduction of WHV cccDNA, WHV DNA RI and WHV RNA from pretreatment level in the low and high dose groups was 16%, 19% and 22% or 25%, 38% and 45%, respectively, indicating that the antiviral effect of SB 9200 was most pronounced for viral RNA. Following the end of treatment, rebound in the hepatic levels of these viral markers was observed in all woodchucks at the end of the study.

### SB 9200 treatment resulted in a decline in hepatic WHV antigen expression and was associated with reduced liver inflammation and transient elevated liver enzyme activity

**Fig 4:**
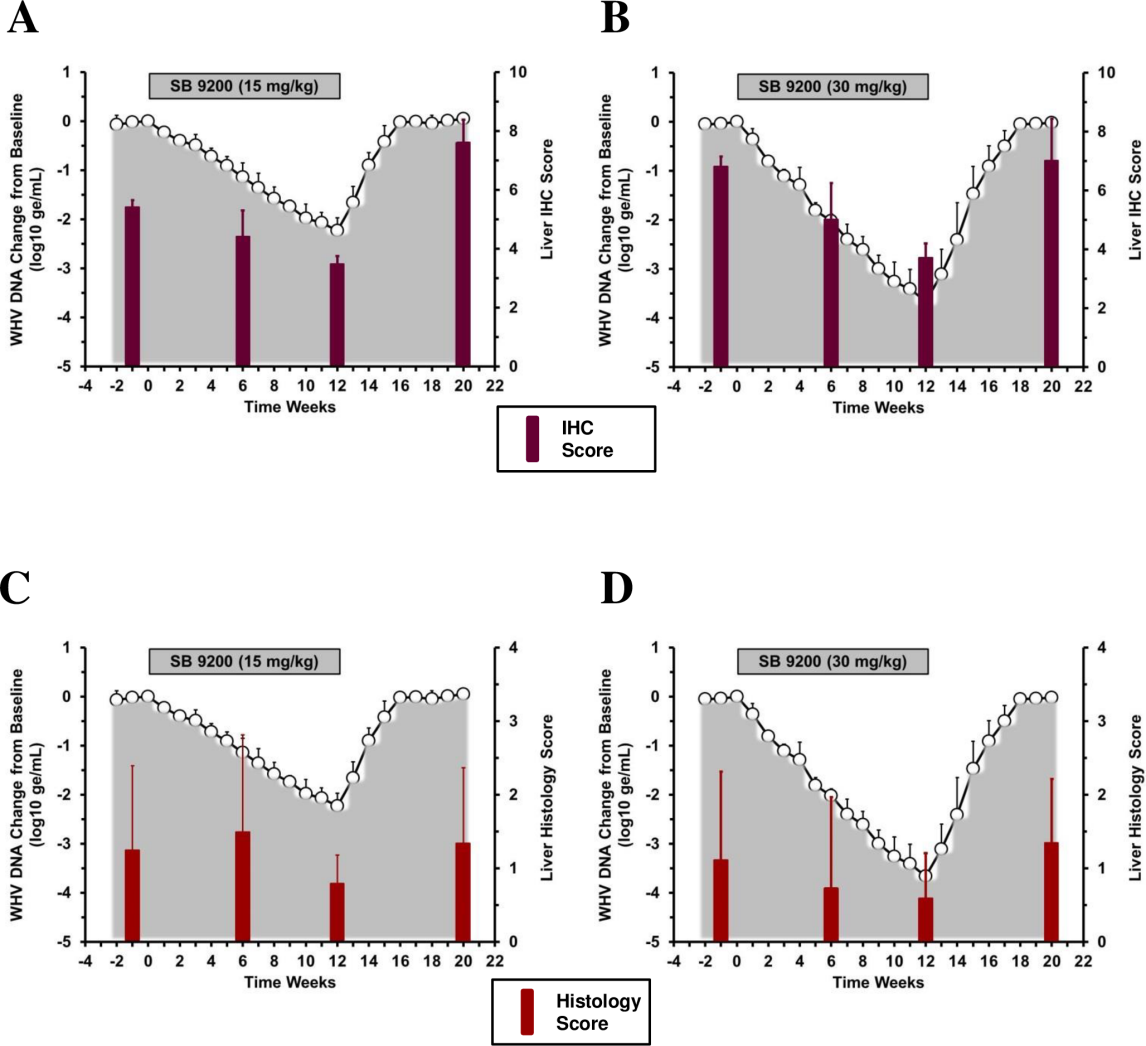
SB 9200 treatment results in transient reduction of WHcAg expression and inflammation in liver. Changes in mean immunohistochemistry (IHC) score for cytoplasmic WHcAg expression in liver (**top panels**) and liver histology score for portal and lobular sinusoidal hepatitis (**bottom panels**) in response to SB 9200 treatment at a low (**A, C**) or high dose (**B, D**). Changes in mean serum WHV DNA relative to T_0_ is plotted on the left y-axis. Error bars represent the standard error of the mean.

SB 9200 treatment caused dose-dependent, transient reductions in hepatic expression scores of cytoplasmic WHcAg from pretreatment level in all woodchucks (Fig 4A and 4B). At the end of treatment, the reductions were most pronounced in animals treated with the higher dose. Following the end of treatment, increases in WHcAg were noted for all woodchucks at the end of the study. Mean WHcAg expression was significantly reduced in the low and high dose groups at weeks 6 and 12 (all *p*<0.05) when compared to pretreatment; however, on a group level the difference was not statistically significant. Comparable reductions were not observed for the expression of cytoplasmic and membranous WHsAg in liver during treatment (data not shown).

There was a trend towards unchanged or even reduced scores of liver inflammation during SB 9200 treatment in both dose groups but variation in individual woodchucks was rather high (Fig 4C and 4D). After the end of treatment, the composite scores for lobular sinusoidal and portal hepatitis increased in most (although not all) animals at the end of the study. Changes in liver inflammation during the 12-week treatment period was comparable in woodchucks of the low and high dose groups, with no statistically significant differences when compared to pretreatment or between the groups.

**Fig 5:**
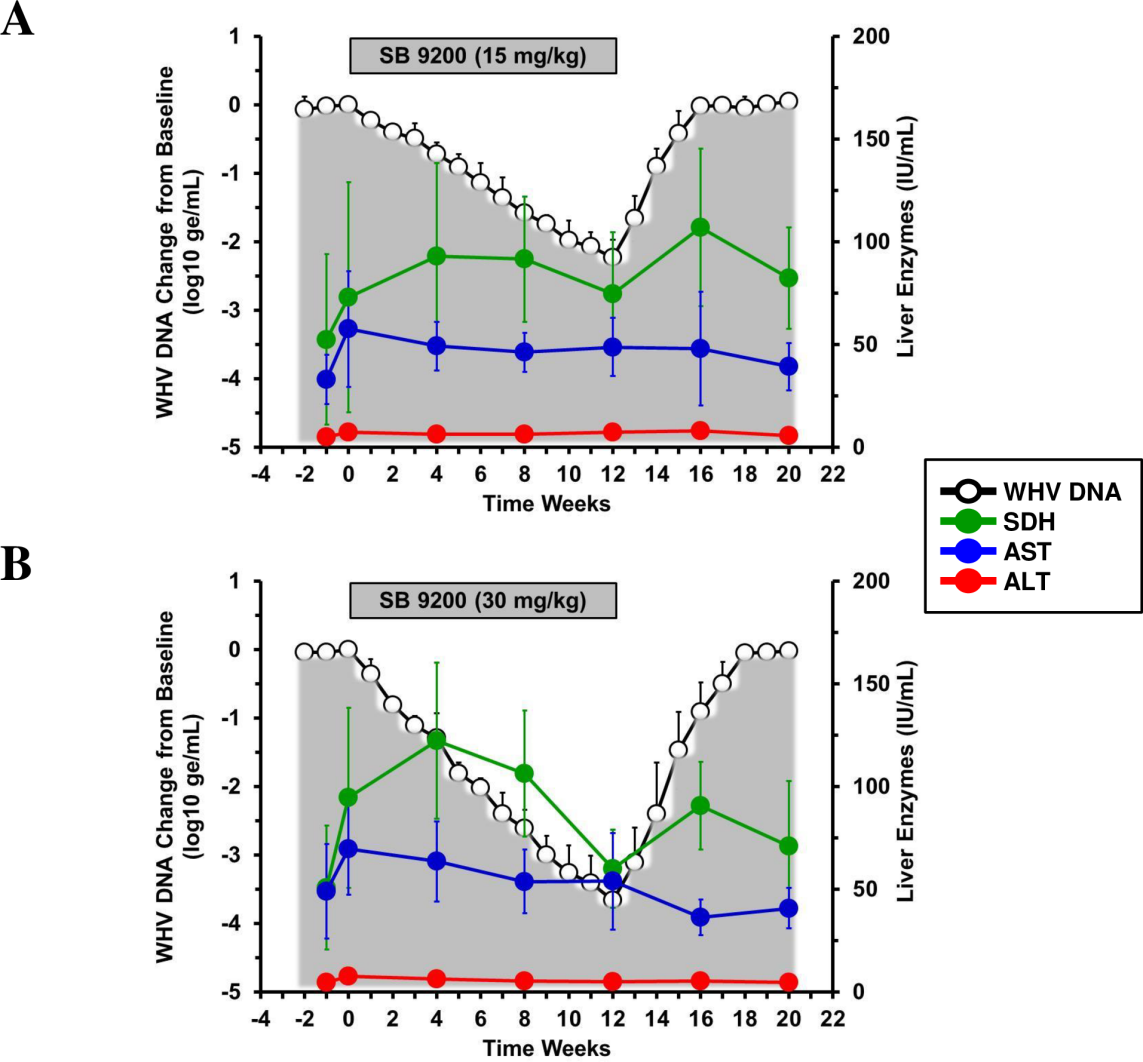
SB 9200 treatment is associated with dose-dependent, transient increases of SDH but not of other liver enzymes. Changes in mean serum levels of SDH, AST and ALT in response to SB 9200 treatment at a low (**A**) or high dose (**B**). Changes in mean serum WHV DNA relative to T_0_ is plotted on the left y-axis. Error bars represent the standard error of the mean.

There was further a trend towards elevated serum SDH levels during SB 9200 administration, especially during the initial 4–8 weeks of treatment, and elevations were more pronounced in the high dose group than in the low dose group (Fig 5). Conversely, at the end of treatment, and at the time of peak antiviral response in both groups, SDH levels declined, and the reductions were again more pronounced in the high dose group. Following the end of treatment, SDH levels became elevated again at the time of initial viral relapse. Serum levels of alanine aminotransferase (ALT) and aspartate aminotransferases (AST) essentially remained unchanged or fluctuated slightly during and following treatment in both groups. On a group level, however, these overall differences were not statistically significant, with the only exception of AST that was significantly reduced in the high dose group during follow-up when compared to pretreatment and treatment levels. Overall, there was a temporal association between peak antiviral response and decline of SDH that also correlated temporally with reduced liver inflammation (see Fig 4C and 4D and Fig 5). This biphasic kinetic of SDH during treatment when correlated to the mounting antiviral response and the declining liver inflammation may be indicative of the host immune response induced by SB 9200.

### SB 9200 treatment induced dose-dependent and sometimes long-lasting expression of type I IFNs and ISGs in blood

**Fig 6:**
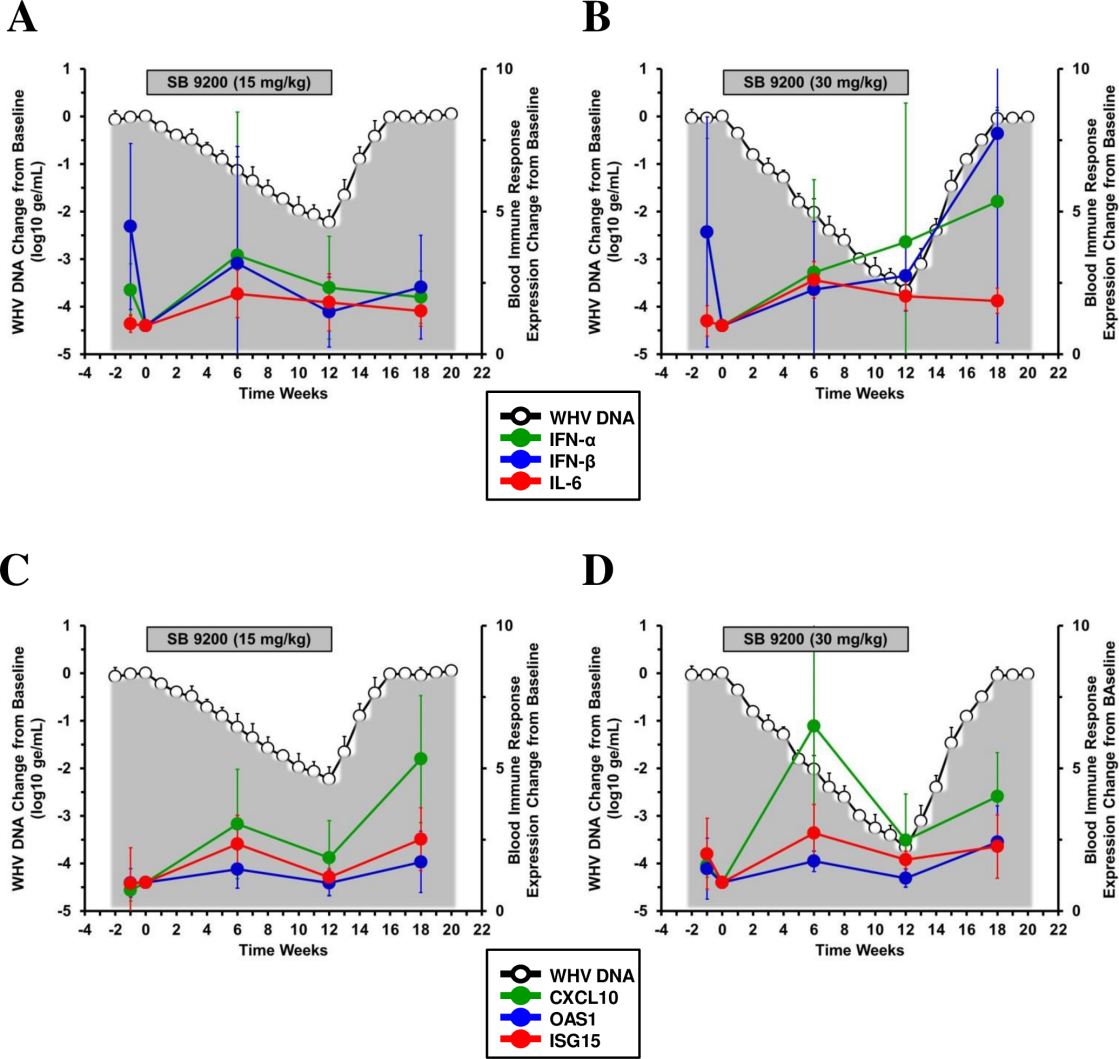
SB 9200 treatment induces dose-dependent and sometimes long-lasting expression increases of type I IFNs, cytokine, and ISGs in peripheral blood. Changes in mean blood transcript levels of IFN-α, IFN-β, and IL-6 (**top panels**) and of CXCL10, OAS1 and ISG15 (**bottom panels**) in response to SB 9200 treatment at a low (**A, C**) or high dose (**B, D**). Changes in mean serum WHV DNA relative to T_0_ is plotted on the left y-axis. Error bars represent the standard error of the mean.

There was a trend of SB 9200 treatment towards the induction of mRNA expression of type I IFNs (i.e., IFN-α and IFN-β) and select antiviral ISGs (i.e., OAS1 and ISG15) in blood, with significant induction at the higher dose when compared to pretreatment (Fig 6). Both the low and high doses also significantly induced the expression of the proinflammatory cytokine, IL-6, and of another ISG, CXCL10, when compared to pretreatment, but increases in transcript level were more pronounced in the high dose group. Transient increases in gene expression were observed at week 6 of treatment in both groups, except for IFN-α and IFN-β in the high dose group as in this case, the expression increased until the end of treatment, and increased further during the follow-up. Conversely, expression of all other genes declined at the end of treatment and stayed at comparable or lower levels during the follow-up, except for the expression of CXCL10 and OAS1 that increased in the low and high dose groups, respectively. However, these overall differences were not statistically significant. In addition, the observed variability in the magnitude of induction of immune response genes did not allow establishing a clear correlation in regard to the magnitude of WHV inhibition observed in both dose groups (see discussion). Since a few (but not all) animals of each dose group had increased expression of IFN-α and/or IFN-β in peripheral blood at week -1 when compared to T_0_, a comparison of basal mRNA expression levels of the above genes in blood of treatment-naïve chronic WHV carrier woodchucks with those of SB 9200 treated animals at pretreatment was performed (S1 Fig), The analysis revealed no difference between the two cohorts of animals and suggests that the elevated expression of type I IFNs and ISGs genes during treatment was drug-mediated. Given that SB 9200 treatment was associated with significant suppression of WHV replication, this suggests that dose-dependent induction of host innate immunity plays a crucial role in the antiviral response mediated by this compound.

### SB 9200 treatment resulted in comparable and long-lasting expression of type I IFNs and ISGs in liver

**Fig 7:**
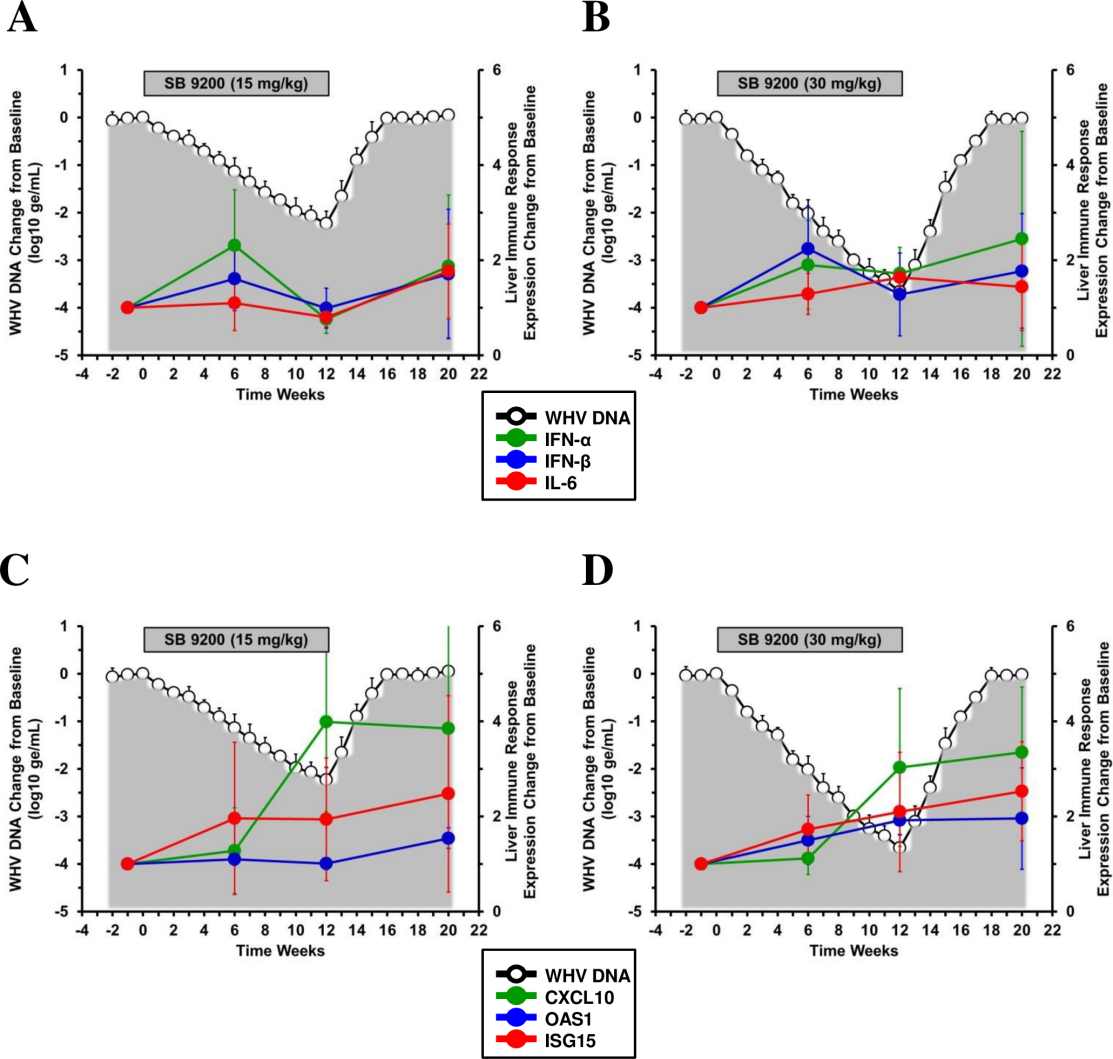
SB 9200 treatment results in comparable and long-lasting expression increases of type I IFNs, cytokine, and ISGs in liver. Changes in mean liver transcript levels of IFN-α, IFN-β, and IL-6 (**top panels**) and of CXCL10, OAS1 and ISG15 (**bottom panels**) in response to SB 9200 treatment at a low (**A, C**) or high dose (**B, D**). Changes in mean serum WHV DNA relative to T_0_ is plotted on the left y-axis. Error bars represent the standard error of the mean.

Analogous to the observations in the periphery, SB 9200 treatment also induced mRNA expression of type I IFNs and ISGs in liver (Fig 7). Compared to pretreatment, expression of IFN-α was significantly induced in the low dose group whereas significantly increased expression of IFN-β, IL-6 and OAS1 was observed in the high dose group. The difference in expression of OAS1 (but not of other genes) was also statistically significant between the low and high dose groups during treatment. Furthermore, CXCL10 expression was significantly induced by treatment in both groups. In contrast to the periphery, the expression of CXCL10, OAS1 and ISG15 in liver was not transient during treatment but increased further through the end of treatment and during the follow-up in both groups. Significant elevation compared to pretreatment was observed at the end of the study for CXCL10 in both groups, for OAS1 in the low dose group, and for ISG15 in the high dose group. There was also a tendency of IL-6 and IFN-α in the low and high dose groups, respectively, towards increased level during the follow-up. The comparison of basal mRNA expression levels of the above genes in liver of treatment-naïve chronic WHV carrier woodchucks with those of SB 9200 treated animals at pretreatment showed again no difference (S2 Fig), indicating that the elevated expression of type I IFN and ISG genes during treatment was drug-mediated. These results suggest that treatment with SB 9200 at two separate doses induces comparable expression of type I IFNs and ISGs in liver. Similar to the periphery, a clear correlation between the magnitude of induction of immune response genes and magnitude of WHV inhibition could not be established (see discussion). As ISG expression lasted beyond the end of treatment and was still elevated during viral relapse, this indicates that additional antiviral immune mechanisms are involved in the treatment response to SB 9200.

### SB 9200 treatment induced long-lasting activation of RIG-I/NOD2 and signaling genes within the IFN pathway and presence of elevated RIG-1 protein levels in liver

**Fig 8:**
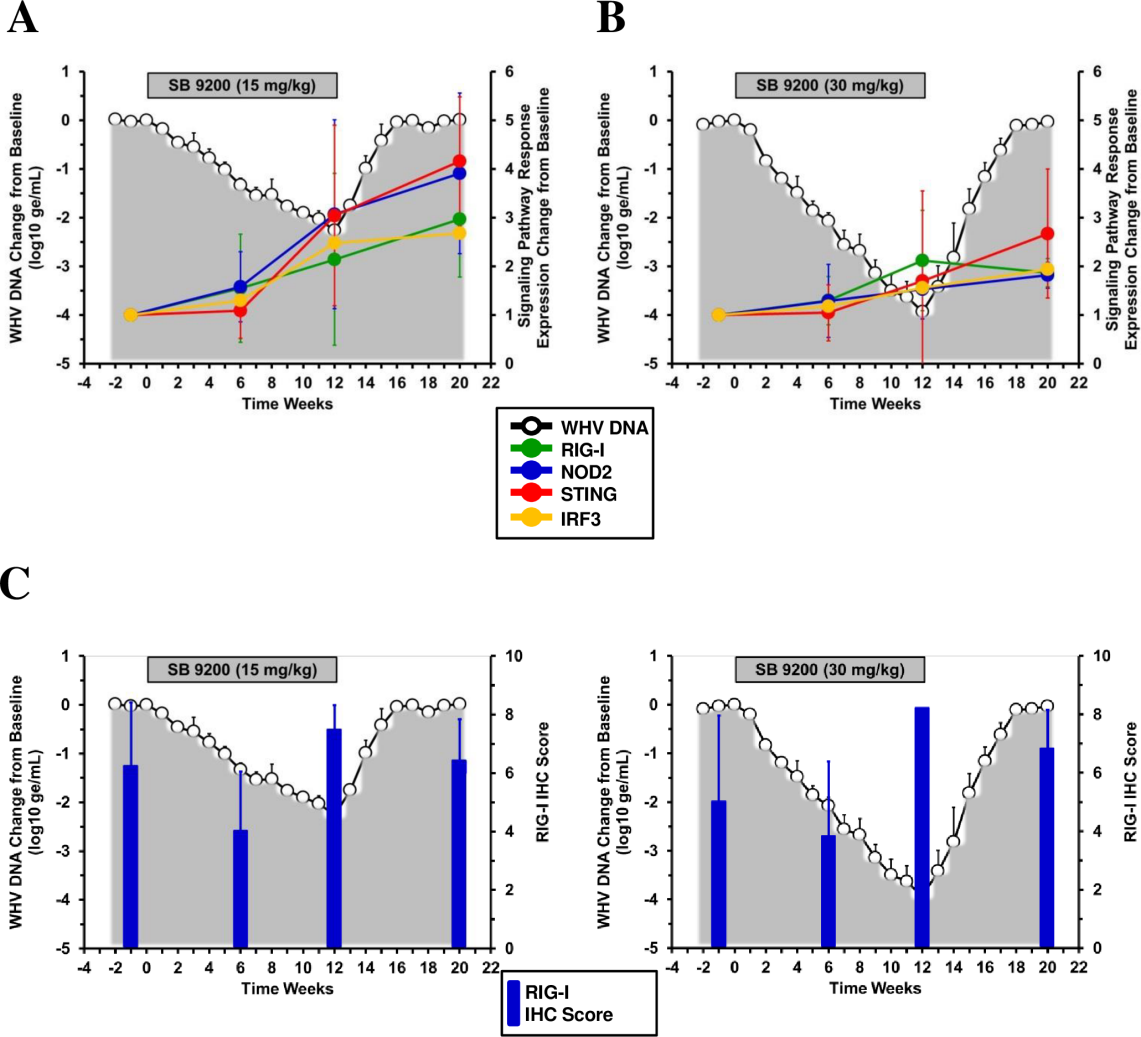
SB 9200 treatment induces long-lasting activation of the RIG-I/NOD2 pathway and presence of elevated RIG-1 protein levels in liver. Changes in mean liver transcript levels of RIG-I, NOD2, STING, and IRF3 in a subset of woodchucks in response to SB 9200 treatment at a low dose (n = 3) (**A**) or high dose (n = 3) (**B**). Changes in mean IHC scores for cytoplasmic RIG-I expression in liver of all woodchucks treated with SB 9200 at a low (n = 5) (**C**) or high dose (n = 5) (**D**). Changes in mean serum WHV DNA relative to T_0_ is plotted on the left y-axis. Error bars represent the standard error of the mean.

In an effort to understand the prolonged induction of innate immune response by SB 9200, the expression of key genes involved in the RIG-I/NOD2 pathway was determined in a subset of woodchucks from both dose groups from which liver biopsies could be collected at all time points during the study. SB 9200 treatment induced the mRNA expression of RIG-I, NOD2, STING, and IRF3 in liver (Fig 8A and 8B). Compared to pretreatment, expression of all genes was significantly induced during treatment and increased further at the end of treatment and during the follow-up. Gene expression was significantly elevated in animals of both groups at the end of the study when compared to treatment but levels were not significantly different between the low and high dose groups. The apparently higher hepatic expression of RIG-I, NOD2, STING, and IRF3 in the low than in the high dose group at the end of treatment and at the end of the study was unexpected but may be explainable by the fact that liver for this additional analysis was only available from a subset of woodchucks of both groups for determination of all study time points. It is conceivable that the timing of peak induction of these genes in individual animals may vary and that the peak transcript levels could not be captured since only selected time points were examined through the study period. However, it is pertinent to mention that elevated expression of STING is consistent with the observed IFN induction by SB 9200 in the liver of woodchucks since the IFN signaling pathways appear to overlap with that of the activation of RIG-1/NOD2 [17]. Overall, these results suggest that type I IFN and ISG gene expression was induced by SB 9200 treatment that correlated with upregulated transcript levels of RIG-I and NOD2. This was further confirmed by SB 9200 treatment induced increases in hepatic expression scores of cytoplasmic RIG-I from pretreatment level in woodchucks from both dose groups (Fig 8C and 8D). As RIG-I levels were increased at the end of treatment in both dose groups when compared to pretreatment levels, and RIG-I stayed elevated through the end of the study in the high dose group, this suggests that SB 9200 treatment results in the long-lasting presence of RIG-I protein in liver.

## Discussion

For establishing efficacy, safety, and pharmacodynamics associated with antiviral response against HBV, chronic WHV carrier woodchucks were treated with SB 9200 for 12 weeks at two separate doses. Treatment was well tolerated and produced dose-dependent and uniform antiviral effects by inducing multi-log reduction in serum WHV DNA and WHsAg and declines in hepatic WHV cccDNA, DNA RI, RNA and WHcAg in all animals.

During infections, viral RNA is mainly sensed by pattern-recognition receptors such as RIG-1 and NOD2 [18, 19]. Binding of these sensor proteins to PAMP within the viral RNA activates downstream signaling pathways which include the mitochondrial antiviral signaling protein (MAVS) leading to the induction of STING, IRF3, IRF7, and NF-κB dependent gene expression, and the subsequent production of type I and type III IFNs and inflammatory cytokines [11, 20]. Thus, sensing of viral RNA is a crucial process to induce antiviral innate immune responses for limiting viral replication and for activation of adaptive immunity [20]. In the case of HBV, it has been recently shown that RIG-I sensing is mediated through recognition of the 5’-end ε region of the HBV pgRNA which leads to the induction of type III rather than type I IFNs in human hepatocytes in response to *in vitro* infection [12]. In addition, activated RIG-I is able to counter-act the interaction of the HBV polymerase with the HBV pgRNA in an IFN pathway independent manner resulting in suppressed viral replication [12]. In this context, it is pertinent to mention that SB 9200 has potent antiviral activity against RNA viruses, including HCV, Norovirus and Respiratory Syncytial Virus [13, 14, 21]. Furthermore, SB 9200 has significant antiviral activity against drug-resistant variants of HBV [7], consistent with the expectation that activation of RIG-I and NOD2 by this compound should be agnostic to the type of virus and genotypes. The present study extended this data as SB 9200 treatment of woodchucks resulted in the prolonged induction of RIG-I, NOD2, STING, and IRF3 expression in liver which correlated with a long-lasting expression of type I IFNs and ISGs in blood and liver. Taken together, the overall data from these studies supports the induction of host innate immune responses by SB 9200 as a significant contributor to its antiviral activity.

Aside from the immune stimulating activity (see below), the antiviral response induced by SB 9200 in the present study was in the range of those of nucleos(t)ide analogs previously evaluated in woodchucks. The magnitude of viral load reduction with SB 9200 was comparable to Emtricitabine, Tenofovir and Adefovir after administration for 12 weeks [15]. However, in contrast to the higher dose of SB 9200, treatment with Emtricitabine and Adefovir did not result in comparable, significant WHsAg reduction. Common for these compounds, as well as SB 9200, was the viral rebound following cessation of treatment. In addition, as SB 9200 did not produce (sustained) loss of WHsAg, seroconversion was not observed.

Comparable to SB 9200, these nucleos(t)ide analogs also induced minor, transient increases in liver enzymes during treatment, before serum activity of SDH (and of AST and/or ALT) became normalized. As elevations in SDH noted during SB 9200 treatment at week 6 were temporally associated with initial reductions in serum WHsAg and hepatic WHV cccDNA, this rise in liver enzyme may indicate immune-mediated viral clearance of infected hepatocytes by cytotoxic effector cells. As SDH activity was transient during treatment and liver inflammation was reduced at the end of treatment, this may further indicate that other, non-cytolytic mechanism(s) contributed to the peak suppression of WHV replication.

As noted previously, the prodrug SB 9200 was rapidly converted to the active metabolite SB 9000 [9, 10] which tends to concentrate in the liver. Consistent with this hypothesis, a dose-dependent correlation between the 2-hour plasma exposure and declines in serum viremia and antigenemia was observed in individual woodchucks. Plasma values seemed to slightly increase with daily dosing during the treatment period. It was recently found that SB 9200/SB 9000 is taken up into hepatocytes by active transport through organic anion-transporting polypeptide (OATP) transporters and enterohepatically recycled consistent with increasing concentration of the compound in the liver over time (Radhakrishnan P. Iyer; personal communication). After the end of treatment, the plasma levels declined to baseline values in all woodchucks indicating clearance of SB 9000. Given these results, it can be speculated that continued dosing beyond 12 weeks may result in much greater suppression of WHV viremia and antigenemia.

As mentioned above, type III rather than type I IFNs are predominantly induced in human hepatocytes in response to *in vitro* HBV infection through RIG-I-mediated sensing [12]. Several studies have suggested that innate immune response in chronic HBV and WHV infections is impaired and that the expression of type I IFNs and of IFN-α and IFN-β stimulated genes is limited in the virus-infected liver [4, 22–24]. Considering the diminished type I IFN response in chronic hepadnavirus infections, the observed peripheral and hepatic induction of IFN-α and IFN-β and of ISGs such as CXCL10, OAS1, ISG15, and the proinflammatory cytokine, IL-6, as well as the induction of RIG-I and NOD2 expression during and even beyond treatment with SB 9200 is important because it suggests that an antiviral innate immune response was induced in a dose-dependent manner.

Since several studies have demonstrated that IFN-α-mediated antiviral effects can directly inhibit HBV and WHV [25–27], an interesting finding of this study was that the antiviral response to SB 9200 did not correlate well with the long-lasting hepatic expression of antiviral ISGs tested, suggesting that other immune response and/or antiviral mechanisms may play a role, especially in the peak response to treatment. This assumption is plausible considering that humans, chimpanzees, and woodchucks resolve HBV and WHV infection without inducing a strong type I IFN response [4, 23, 28]. However, there are several limitations to consider. Since only three antiviral ISGs were tested, peripheral and hepatic expression of other ISGs may be important to and correlate with treatment response. Furthermore, the durable expression of antiviral ISGs beyond the end of treatment may not entirely attributable to SB 9200 and could additionally include an immune response of the host to the recurrence of viral replication following cessation of treatment. It is further of note that type III IFN (i.e., IFN-λ) that is predominantly produced *via* RIG-I-mediated sensing during *in vitro* HBV infection [12] was apparently not induced in blood and liver during SB 9200 treatment in woodchucks (data not shown). However, since gene expression during treatment was restricted to 2–4 hours post-dose, it is likely that maximum expression of immune response genes was missed and that peak induction may be associated with the antiviral response to SB 9200. As individual woodchucks showed peak induction of IFNs and ISGs either at week 6 or week 12 of SB treatment, variation in the gene expression in both dose groups was rather high during the treatment period. This variability, with relatively low magnitudes of 2–5 fold above baseline, and with standard errors sometimes exceeding the mean of gene expression, did not allow finding a clear correlation between magnitude of induction of immune response genes and magnitude of WHV inhibition even given that transcript levels of IFNs and ISGs in both blood and liver were examined. In fact, the two woodchucks of the high dose group with the most pronounced declines in viremia and antigenemia (F3027 and F3030) showed highly variable induction during treatment (one animal had high expression of immune response genes while the other showed only minor increased transcript levels during or at the end of treatment; data not shown). One possible explanation is that a lag phase between the induction of IFNs and ISGS and antiviral response is present. In clinical settings, it has been observed that HCV-infected patients while on IFN therapy show a variable antiviral response with an initial reduction of viral load over three days followed by a much slower decline after intravenous administration of IFN [29]. Hence, even in the case of exogenously administered IFN, there is a delay in antiviral response to the time of administration unlike in the case of direct acting antivirals where antiviral response occurs within several hours of administration. Furthermore, in a recent seven-day clinical trial of SB 9200 in HCV-infected patients, antiviral effects similar to that of IFN in terms of both induction and extent of antiviral activity were observed, and a good correlation between peak plasma level of SB 9200, viral RNA decline and induction of IFN, ISG15, and OAS1 in responders *versus* non-responders was established (Radhakrishnan P. Iyer; personal communication). As opposed to the present study in woodchucks, the plasma levels, immune parameters, and HCV load decline during this clinical trial could be measured at several different time points whereby allowing to demonstrate this correlation. Additional studies will be required to determine in greater detail the panel of intrahepatic innate immune response genes and to correlate with the antiviral response induced by SB 9200.

The apparent inconsistency between antiviral effects of SB 9200 in periphery (3.7 log_10_ and 1.6 log_10_ reduction in serum WHV DNA or WHsAg, respectively) and liver (25–45% reduction in hepatic WHV nucleic acids) as observed in the high dose group can be likely explained by the complex pharmacokinetics and the proposed mechanism of action of SB 9200. Specifically, maximal reductions in hepatic WHV replication may not necessarily correlate with maximal declines in serum viremia. In addition, the reduction in hepatic expression of viral nucleic acids is dependent on the ability of the immune-mediated mechanism to either impact transcription of cccDNA or to reduce cccDNA levels both of which may require duration of treatment longer than 12 weeks. Indeed, in infected hepatocytes, reduction in cccDNA is dependent on hepatocyte turn-over which has a half-life of several days [30]. Considering all the data derived from the present study, and in analogy to the mechanism described for HBV [12], it appears that SB 9200 has also a direct antiviral component that may involve interference (i.e., steric blockage) of the WHV polymerase to engage with WHV pgRNA by SB 9200 activated RIG-I and NOD2, and that may have contributed to the overall, and especially peak treatment response. The assumption is consistent with the demonstrated potent efficacy of SB 9200 in HBV transgenic mice, an inherently immunotolerant animal model of chronic HBV infection. In a dose-ranging study in this model, daily oral administration of SB 9200 for 14 days resulted in significant reduction in liver HBV DNA and the achieved antiviral effect at higher doses was comparable to that of Adefovir [8].

In summary, by establishing the efficacy, safety, and pharmacodynamics of SB 9200 in an immunocompetent animal model of CHB, this study provided insights into the immune stimulating and direct antiviral activities of this new class of anti-HBV compounds. These findings have important implications for further development of SB 9200 for therapy of CHB, and also provide rationale for evaluating combination treatment of SB 9200 with approved anti-HBV nucleos(t)ide analogs in the woodchuck for inducing functional cure of chronic WHV infection, and by analogy of chronic HBV infection in patients.

## Supporting Information

S1 Fig Comparison of basal expression levels of type I IFNs, cytokine, and ISGs in peripheral blood of age-matched, treatment-naïve chronic WHV carrier woodchucks with pretreatment levels in SB 9200 treated woodchucks. Mean levels of blood transcripts of IFN-α (**A**), IFN-β (**B**), IL-6 (**C**), CXCL10 (**D**), OAS1 (**E**), and ISG15 (**F**) in five untreated control woodchucks (Control) and in ten woodchucks of the combined low and high dose groups (LD + HD). Transcript levels of host innate immune response genes for woodchucks of the low and high dose groups were obtained at T_0_ (pretreatment baseline). The bar height indicates the mean of each group. The *p*-values above the horizontal lines indicate the level of statistical significance between groups.
(PPTX)

S2 Fig Comparison of basal expression levels of type I IFNs, cytokine, and ISGs in liver of age-matched, treatment-naïve chronic WHV carrier woodchucks with pretreatment levels in SB 9200 treated woodchucks. Mean levels of liver transcripts of IFN-α (**A**), IFN-β (**B**), IL-6 (**C**), CXCL10 (**D**), OAS1 (**E**), and ISG15 (**F**) in five untreated control woodchucks (Control) and in ten woodchucks of the combined low and high dose groups (LD + HD). Transcript levels of host innate immune response genes for woodchucks of the low and high dose groups were obtained at week -1 (pretreatment baseline). The bar height indicates the mean of each group. The *p*-values above the horizontal lines indicate the level of statistical significance between groups.
(PPTX)
