# Supplementary figures and images for: Antiviral Efficacy and Host Innate Immunity Associated with SB 9200 Treatment in the Woodchuck Model of Chronic Hepatitis B
Source: PLoS One. 2016 Aug 23;11(8):e0161313. doi: 10.1371/journal.pone.0161313 (PMC4995001; doi:10.1371/journal.pone.0161313)

## Slide 1
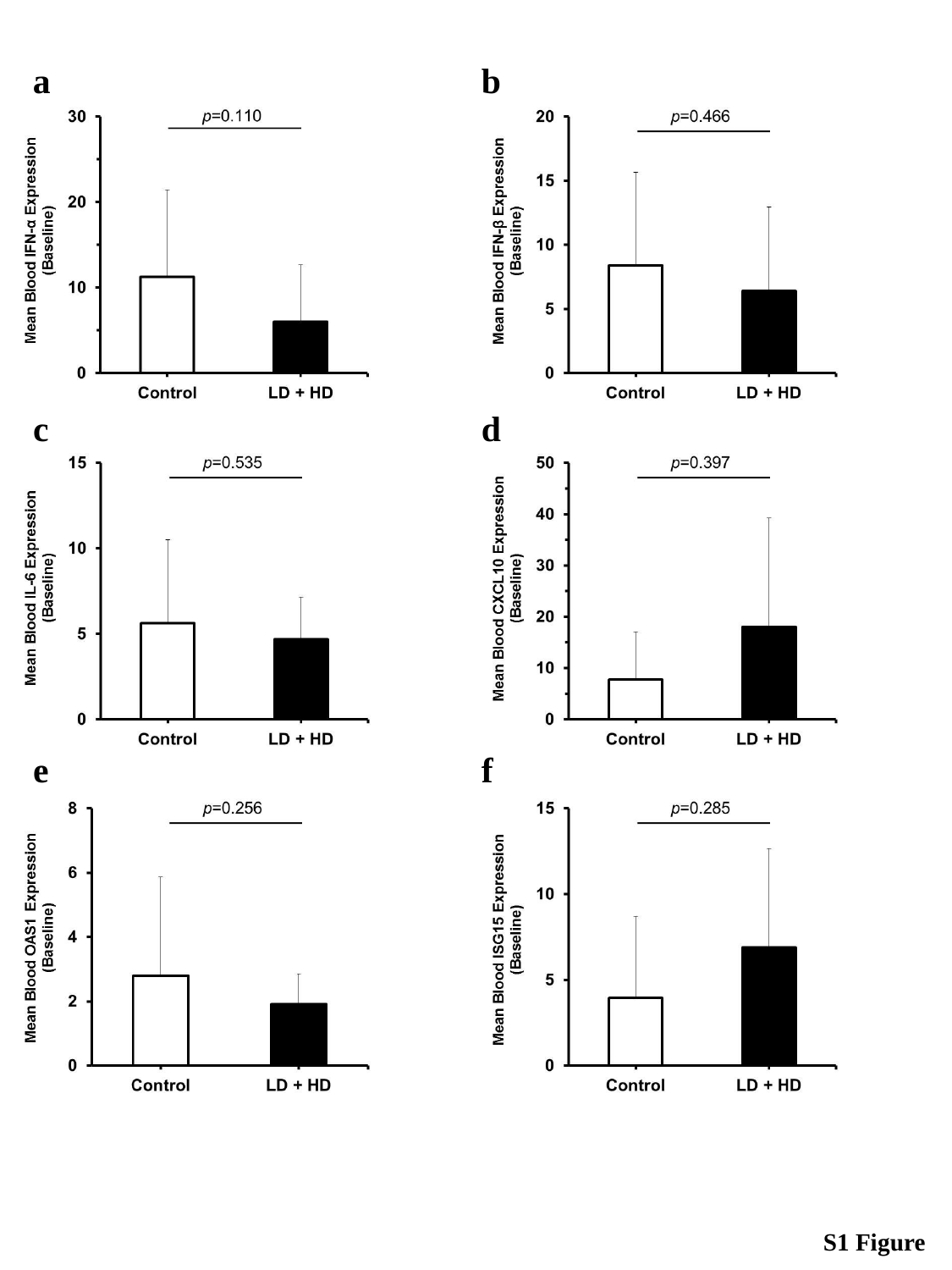

a
 c
 e
 b
 d
 f
S1 Figure

Supplement: S1 Fig — Mean levels of blood transcripts of IFN-α (A), IFN-β (B), IL-6 (C), CXCL10 (D), OAS1 (E), and ISG15 (F) in five untreated control woodchucks (Control) and in ten woodchucks of the combined low and high dose groups (LD + HD). Transcript levels of host innate immune response genes for woodchucks of the low and high dose groups were obtained at T0 (pretreatment baseline). The bar height indicates the mean of each group. The p-values above the horizontal lines indicate the level of statistical significance between groups. (PPTX) [file pone.0161313.s001.pptx]

## Slide 1
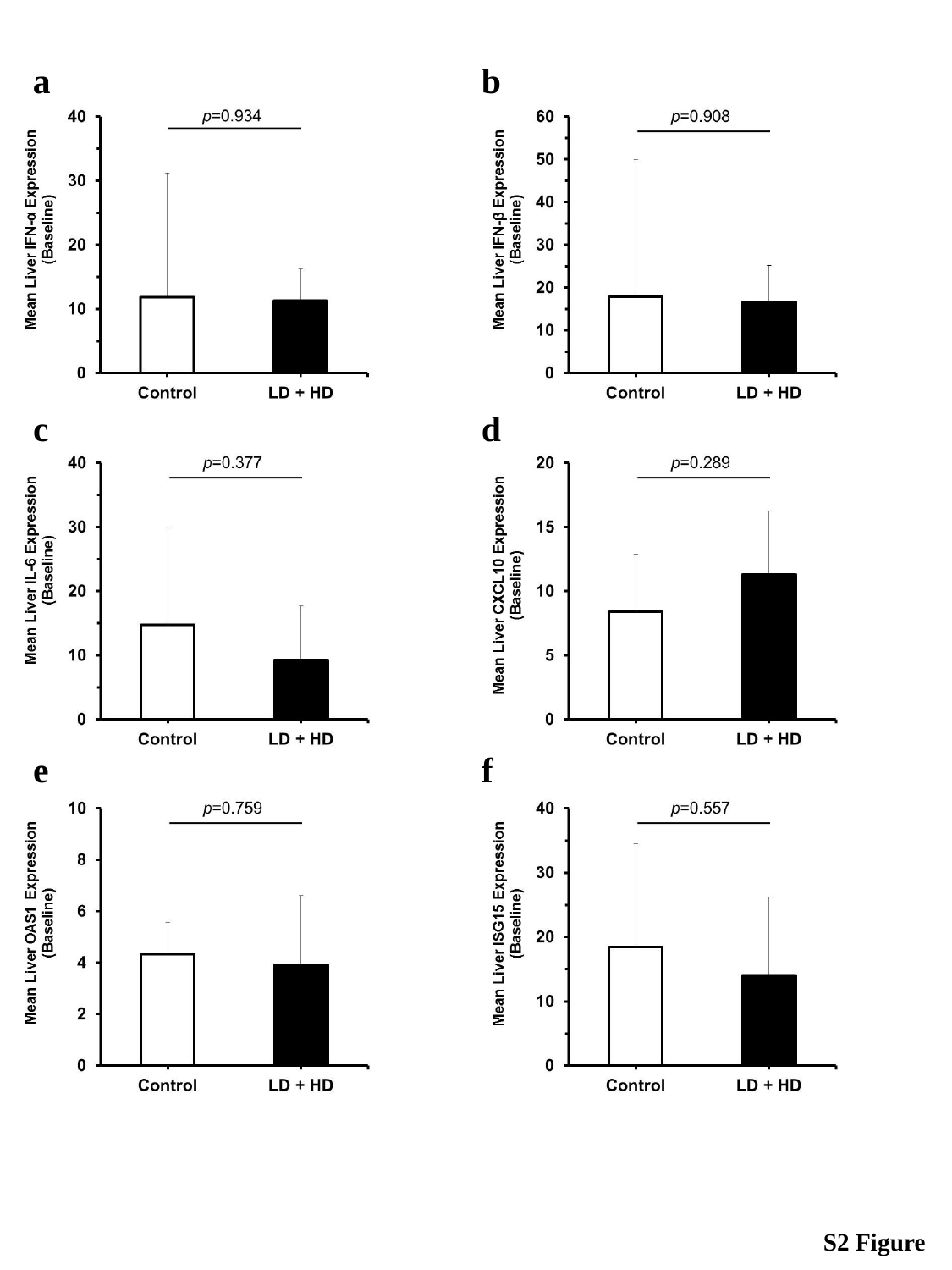

a
 c
 e
 b
 d
 f
S2 Figure

Supplement: S2 Fig — Mean levels of liver transcripts of IFN-α (A), IFN-β (B), IL-6 (C), CXCL10 (D), OAS1 (E), and ISG15 (F) in five untreated control woodchucks (Control) and in ten woodchucks of the combined low and high dose groups (LD + HD). Transcript levels of host innate immune response genes for woodchucks of the low and high dose groups were obtained at week -1 (pretreatment baseline). The bar height indicates the mean of each group. The p-values above the horizontal lines indicate the level of statistical significance between groups. (PPTX) [file pone.0161313.s002.pptx]
